# Infection of Porcine Circovirus 2 (PCV2) in Intestinal Porcine Epithelial Cell Line (IPEC-J2) and Interaction between PCV2 and IPEC-J2 Microfilaments

**DOI:** 10.1186/s12985-014-0193-0

**Published:** 2014-11-19

**Authors:** Mengfei Yan, Liqi Zhu, Qian Yang

**Affiliations:** College of Veterinary Medicine, Nanjing Agricultural University, Nanjing, 210095 PR China

**Keywords:** PCV2, Intestinal epithelial cell, Microfilaments

## Abstract

**Background:**

Porcine circovirus-associated disease (PCVAD) is caused by a small pathogenic DNA virus, *Porcine circovirus* type 2 (PCV2), and is responsible for severe economic losses. PCV2-associated enteritis appears to be a distinct clinical manifestation of PCV2. Most studies of swine enteritis have been performed in animal infection models, but none have been conducted *in vitro* using cell lines of porcine intestinal origin. An *in vitro* system would be particularly useful for investigating microfilaments, which are likely to be involved in every stage of the viral lifecycle.

**Methods:**

We confirmed that PCV2 infects the intestinal porcine epithelial cell line IPEC-J2 by means of indirect immunofluorescence, transmission electron microscopy, flow cytometry and qRT-PCR. PCV2 influence on microfilaments in IPEC-J2 cells was detected by fluorescence microscopy and flow cytometry. We used Cytochalasin D or Cucurbitacin E to reorganize microfilaments, and observed changes in PCV2 invasion, replication and release in IPEC-J2 cells by qRT-PCR.

**Results:**

PCV2 infection changes the ultrastructure of IPEC-J2 cells. PCV2 copy number in IPEC-J2 cells shows a rising trend as infection proceeds. Microfilaments are polymerized at 1 h p.i., but densely packed actin stress fibres are disrupted and total F-actin increases at 24, 48 and 72 h p.i. After Cytochalasin D treatment, invasion of PCV2 is suppressed, while invasion is facilitated by Cucurbitacin E. The microfilament drugs have opposite effects on viral release.

**Conclusion:**

PCV2 infects and proliferates in IPEC-J2 cells, demonstrating that IPEC-J2 cells can serve as a cell intestinal infection model for PCV2 pathogenesis. Furthermore, PCV2 rearranges IPEC-J2 microfilaments and increases the quantity of F-actin. Actin polymerization may facilitate the invasion of PCV2 in IPEC-J2 cells and the dissolution of cortical actin may promote PCV2 egress.

## Introduction

*Porcine circovirus* (PCV), a member of the family *Circoviridae*, is the smallest known non-enveloped, single-stranded, circular DNA virus [1]. Two types of PCV are now recognized. PCV1, originally identified as a contaminant of the porcine kidney cell line PK-15, is considered to be nonpathogenic [2-4] although it can replicate efficiently and produce pathology in the lungs of porcine foetuses [5]. PCV2 is associated with porcine circovirus-associated disease (PCVAD), which was first described in the early 1990s and has since emerged as an economically damaging disease worldwide [6]. PCVAD causes diverse pathologies, including porcine multisystemic wasting syndrome (PMWS), porcine dermatitis and nephropathy syndrome (PDNS), porcine respiratory disease complex (PRDC), reproductive failure, acute pulmonary edema (APE), and other diseases [7-11]. This article focuses on enteritis, another important clinical manifestation [12-14]. The oronasal route is considered the most likely route of PCV2 infection [15] but the key mechanisms involved in infection, especially the invasion, replication and release of PCV2, have not been studied *in vitro.*

Actin is crucial for many kinds of important cellular processes including cell division, polarity, motility, uptake of nutrients and intracellular transport. Actin occurs in two basic forms, monomeric or globular actin (G-actin) and filamentous actin (F-actin). Cell biologists have used small-molecule modulators of actin to manipulate the cytoskeleton. For example, Cytochalasin D (CytD) inhibits actin polymerization, blocking the addition of new monomers to actin filaments by binding to the positive end of actin filaments [16]. Cucurbitacin E is a potent inhibitor of actin depolymerization, binding specifically to F-actin by forming a covalent bond at residue Cys257 [17].

Microfilaments formed by F-actin fibres present both a powerful barrier and a potential weakness for viral invasion. Interactions between influenza virus and the actin cytoskeleton have been observed throughout the virus life cycle, including entry [18], intracellular trafficking [19] and egress [20]. PCV2 DNA impairs actin polymerization in plasmacytoid dendritic cells (DCs) and endocytosis in monocyte-derived DCs [21], potentially explaining PCV2-induced immunosuppression [22]. PCV2 internalization in porcine kidney (PK-15), swine kidney (SK) and swine testicle (ST) epithelial cells is mediated by actin and Rho-GTPase [23]. Here we examine the link between PCV2 and microfilaments in the intestinal porcine epithelial cell line, IPEC-J2, and develop an intestinal cell infection model for investigating PCV2 pathogenesis.

## Materials and methods

### Cells and virus

IPEC-J2 cell lines free from *Porcine circovirus* (Guangzhou Jennio Biotech Co.,Ltd., China) were used in this study. IPEC-J2 cells were cultured in Dulbecco’s Modified Eagle’s Medium nutrient mixture F-12 (DMEM/F-12 from Life Technologies, USA) supplemented with 5% fetal bovine serum (FBS, Life Technologies, USA), 16 mM HEPES (Life Technologies, USA) and 5 ng/mL epidermal growth factor (EGF, BD Biosciences, Germany), and incubated in an atmosphere of 5% CO_2_ at 37°C [24]. Cells were routinely seeded at a density of 2 × 10^5^/mL in plastic tissue culture flasks (25 cm^2^ flasks, Corning, USA) and passaged every 3–4 days for a maximum of 20 times. In our experiments, IPEC-J2 cells were grown on 6- or 24-well plastic tissue culture plates (Corning, USA) at a density of 3 × 10^5^/well or 1.5 × 10^6^/well, respectively.

PCV2 strain WG09 (GenBank accession no. GQ845027) was kindly provided by Professor Ping Jiang [25]. The virus stock was a fourth-passage cell culture prepared in PK-15 cells with a titer of 10^6^ TCID_50_/ml.

### Virus titration by IFA

To determine the infectious titer of PCV2 virus stock in IPEC-J2 cells, cells were cultivated on coverslips in 24-well tissue culture plates. Virus stock was serially diluted 10-fold in DMEM/F-12, and each dilution was inoculated onto 10 wells containing IPEC-J2 cell monolayers. Wells containing mock infected cells were included as controls. Infected cells were fixed at 3 days post-inoculation with 4% paraformaldehyde in 0.01 M PBS buffer at room temperature for 20 min. After washing with PBS buffer, infected cells were incubated with a 1:500-diluted PCV2 capsid protein rabbit polyclonal antibody (Global Biotech, USA) at 37°C for 1 h. The cells were then washed three times with PBS buffer and incubated with a DyLight488 goat anti-rabbit IgG secondary antibody (Liankebio, China) at 37°C for 45 min. Finally, the cells were washed, stained 5 min with DAPI (diluted 1000-fold, Life Technologies, USA) rinsed again then mounted on microslides and examined under a fluorescence microscope (ZEISS Observer.Z1, Germany). Five microscope fields per coverslip were selected to calculate the 50% tissue culture infective dose (TCID_50_) per ml.

### Transmission electron microscopy

IPEC-J2 cells were grown on 6-well tissue culture plates and infected with PCV2 at 3 × 10^2.5^ TCID_50_/ml for 1 and 48 h. Wells containing mock infected cells were included as controls. Cells at various times were fixed with 2.5% glutaraldehyde in 0.1 M PBS buffer for 3 h at 4°C. Subsequently, samples were processed as described [26] and analyzed by using a Hitachi-7650 transmission electron microscope (TEM, Japan) at 120 kV.

### Flow cytometry

IPEC-J2 cells were grown on 6-well tissue culture plates and infected with PCV2 at 3 × 10^2.5^ TCID_50_/ml for 1, 24, 48 and 72 h. Wells containing mock infected cells were included as controls. Cells at various times were harvested and cultured at 37°C with a PCV2 capsid protein rabbit polyclonal antibody and a DyLight488 goat anti-rabbit IgG secondary antibody as described above. After antibody incubation, cells were washed and the viral mean fluorescence intensity (MFI) was determined using a FACS Calibur flow cytometer (BD, USA). For flow cytometric analyses, three replicas are presented.

### Viral growth curve by qRT-PCR

To determine PCV2 virus loads in cells and supernatants collected from PCV2-infected IPEC-J2 cells, cells were grown on 24-well tissue culture plates and infected with PCV2 at 3 × 10^2.5^ TCID_50_/ml for 6, 12, 24, 48, 72, 96 and 120 h. Wells containing mock infected cells were included as controls. The supernatants at various times post-infection were transferred directly into centrifuge tubes, while cells were harvested by trypsin digestion and then transferred into centrifuge tubes. The volume of both supernatants and trypsin-treated cells was 200 μl. Viral DNAs were isolated from supernatants or cells using a Mag-Bind Viral DNA/RNA Kit (Omega Bio-Tek, USA) according to the manufacturer’s instructions. The DNAs extracted from the cells and/or cell culture supernatants were resuspended in 50 μl of DNase-, RNase-, and proteinase-free water.

For PCR, primers containing restriction sites for Hind III and Kpn I were designed to amplify a 447-bp ORF2 fragment of PCV2 (sense primer 5′-GTCCTGGTCGTATTTACTGTTT-3′; antisense primer 5′-GTCAGAACGCCCTCCTG-3′). The amplicon was cloned into the pMD®19-T Simple Vector (Takara, Japan). The recombinant plasmid was purified and quantitated by optical density (OD_260_). Ten-fold dilutions were prepared to provide 10^9^–10^2^ plasmids per 2 μl sample for PCR. The dilutions were stored at −20°C, while plasmid stocks were stored at −70°C.

qPCR was performed in a 25 μl reaction volume using an ABI 7500 thermocycler (Applied Biosystems, USA). For supernatant and cell samples, 2 μl of the DNA eluate was used as template. The real-time PCR procedure followed the protocol provided with SYBR® Premix Ex Taq™ GC (Takara, Japan). Three concentrations of recombinant plasmid (10^8^, 10^5^, and 10^2^ copies per sample) were included in each run as positive controls, and were used derive the standard curve for quantitation of PCV2 DNA in supernatant and cell samples. Each run included two negative controls (no template).

### Microfilament changes by fluorescence microscopy

IPEC-J2 cells were grown on coverslips in 24-well tissue culture plates and infected with PCV2 at 3 × 10^2.5^ TCID_50_/ml for 1, 24, 48 and 72 h. Wells of mock infected cells were included as controls. Cells were treated following the protocol recommended for Alexa Fluor 488 phalloidin (Life Technologies, USA). DAPI (Life Technologies, USA) was used as a nuclear counterstain. Finally, cells were mounted on microslides and fluorescence images were recorded using a ZEISS Observer.Z1.

### Microfilament changes by FCM

IPEC-J2 cells were cultivated on 6-well tissue culture plates and infected with PCV2 at 3 × 10^2.5^ TCID_50_/ml for 1, 24, 48 and 72 h. Wells of mock infected cells were included as controls. Cells at various times were harvested and cultured with Alexa Fluor 488 phalloidin as described in section 2.6. After phalloidin incubation, cells were washed and the F-actin MFI was analyzed on a FACS Calibur flow cytometer (BD, USA). For flow cytometric analyses, three replicas are presented.

### Viral lifecycle change after CytD/CuE by RT-PCR

Microfilament drug stocks were prepared in DMSO at concentrations of 4 mM for CytD (Life Technologies, USA) and 27 mM for CuE (Sigma-Aldrich, USA). Different dilutions were assessed to determine concentrations with maximal effects on microfilaments without losses in cell viability, based on instructions accompanying the cell counting Kit-8 (Beyotime, China). In all experiments the concentration of CytD was 2 μM and the concentration of CuE was 50 nM. Drugs were diluted to final concentrations in cell culture medium and added to infected IPEC-J2 cells. Specifically, for investigating changes due to viral invasion, cells were first treated with CytD/CuE for 2 h, then infected with PCV2 at 3 × 10^2.5^ TCID_50_/ml. After 1 h, cells were harvested for quantitation of PCV2 DNA. For investigating changes in viral replication and release, cells were first infected with PCV2 at 3 × 10^2.5^ TCID_50_/ml for 1 hr, then treated with CytD/CuE, these cells and supernatants were harvested at 24, 48 and 72 h p.i. PCV2 DNA was quantitated in both cells and supernatants for replication, but in supernatants only for release. Infected cells untreated with CytD/CuE were included as controls. The quantitation of PCV2 DNA was accomplished as described earlier.

### Statistical analysis

Statistical analysis was performed using Statistical Program for Social Sciences (SPSS) 16.0. Significance was determined by Analysis of Variance (ANOVA). A *P* value less than 0.05 was considered to be significant, and less than 0.01 was considered to be highly significant.

## Results

### Viral appearance in cells

**Figure 1:**
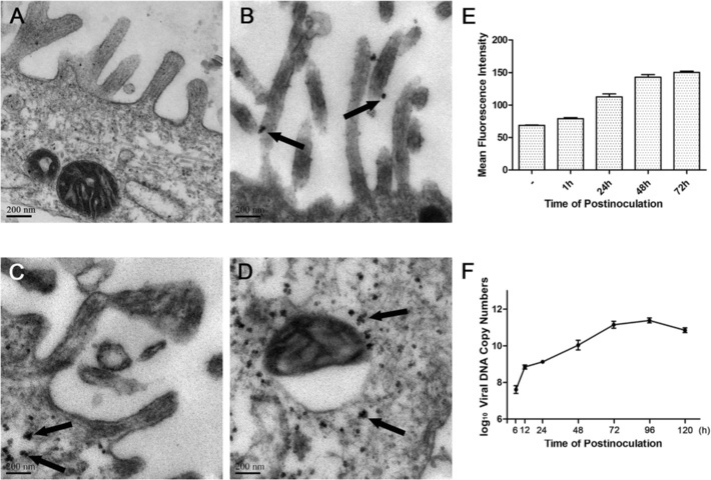
**Infection of PCV2 in IPEC-J2 Cells. (A, B, C and D).** Electron microscopic analysis of ultrathin sections of IPEC-J2 cells either without PCV2 (A , control ), or infected with PCV2 at 3 × 10^2.5^ TCID_50_/ml for 1 h **(B)** and 48 h **(C and D)**. Black arrows indicate virus. **(E)** Flow cytometry analysis of mean fluorescence intensity of PCV2 in IPEC-J2 cells either without PCV2 (− , control ), or infected with PCV2 at 3 × 10^2.5^ TCID_50_/ml for 1, 24, 48 and 72 h. PCV2 was detected using a PCV2 capsid protein rabbit polyclonal antibody and a DyLight488 goat anti-rabbit IgG secondary antibody. Values are means ± SEM for three separate experiments. **(F)** Viral growth curve. IPEC-J2 cells were infected with PCV2 at 3 × 10^2.5^ TCID_50_/ml. Supernatants and cells were harvested at 6, 12, 24, 48, 72, 96 and 120 h p.i. To obtain cell-associated infectivity, viral DNAs were quantitated by qRT-PCR assay. Values are means ± SEM for three separate experiments.

The infectious titer of the PCV2 virus stock prepared from IPEC-J2 cells was determined to be 10^4.5^ TCID_50_/ml. In a survey using TEM, virus appeared near microvilli of differing lengths and widths at 1 h p.i. (Figure 1B; compare uninfected cells in Figure 1A) and in cells at 48 h p.i. (Figure 1C and D). Mitochondria increased in size and became more spherical, mitochondrial matrixes became shallower, and cristae were shortened and reduced in number. Mitochondrial vacuolization was also observed at 48 h p.i. (Figure 1D).

### Viral proliferation in cells

Fluorescently labeled viruses in PCV2-infected cells were detected using FCM. As shown in Figure 1E, viral MFI increased from 1 to 72 h p.i. Similarly, total viral DNAs detected by qRT-PCR in PCV2-infected cells and supernatants increased from 6 to 96 h p.i., although DNA decreased at 120 h p.i. (Figure 1F). These data demonstrate that PCV2 can proliferate in IPEC-J2 cells.

### Microfilament changes by virus

**Figure 2:**
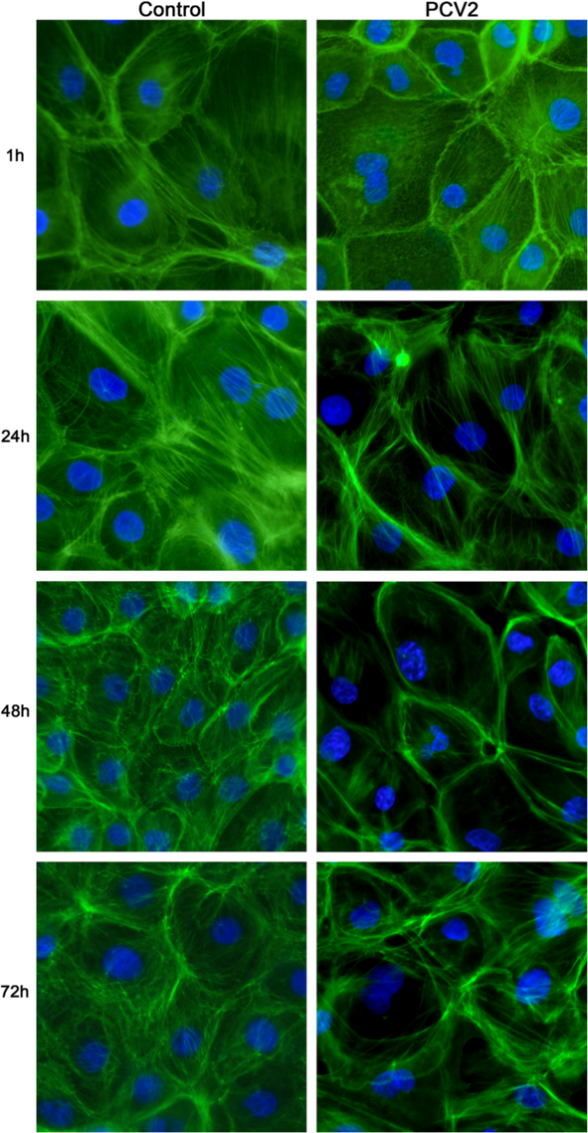
**Microfilament changes observed by fluorescence microscopy.** IPEC-J2 cells were infected with PCV2 at 3 × 10^2.5^ TCID_50_/ml. For actin staining at 1, 24, 48 and 72 h p.i., cells were fixed with paraformaldehyde and permeabilized with Triton X-100. F-actin was detected by phalloidin-FITC. DAPI was used as a nuclear counterstain. Images were obtained using a fluorescence microscope (ZEISS Observer.Z1). Magnification 400x.

**Figure 3:**
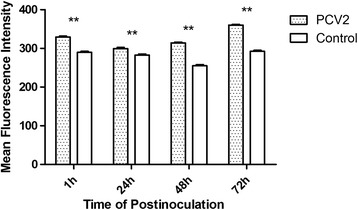
**Microfilament changes detected by FCM.** Flow cytometry analysis of mean fluorescence intensity of F-actin in IPEC-J2 cells infected with PCV2 at 3 × 10^2.5^ TCID_50_/ml for 1, 24, 48 and 72 h. F-actin was detected using phalloidin-FITC. Asterisks indicate highly significant differences between experimental and control samples (*P* <0.01) (n = 3).

To determine whether PCV2 affects F-actin during infection, IPEC-J2 cells were fluorescently stained at different times post infection and examined by fluorescence microscopy. Uninfected IPEC-J2 cells were typically cobblestone in shape with clear boundaries, and contained intra-cytoplasmic microfilaments scattered in a parallel formation. In contrast, microfilaments in infected cells were distributed and granular in appearance at 1 h p.i., although weak parallel patterns could be discerned in some cases. At 24 h p.i., perinuclear microfilaments were greatly reduced and cell boundaries became clear in the PCV2-infected cells. Infected cells at 48 h p.i. were similar to those at 24 h p.i. At 72 h p.i., microfilament disorganization under the plasma membrane was apparent, but some reorganization could be observed below the plasma membrane in the infected cells (Figure 2). Changes in total cellular F-actin in PCV2-infected cells were also determined using FCM. As shown in Figure 3, total F-actin in both infected and uninfected cells decreased at 24 and 48 h p.i. and then increased at 72 h p.i., compared to levels at 1 h p.i. However, the MFI of F-actin in infected cells was higher than the MFI of F-actin in uninfected cells at all observed time points. The microscopic evaluation and F-actin assays demonstrate that PCV2 influences microfilaments both morphologically and quantitatively in IPEC-J2 cells.

### Viral life cycle changes in response to CytD/CuE

**Figure 4:**
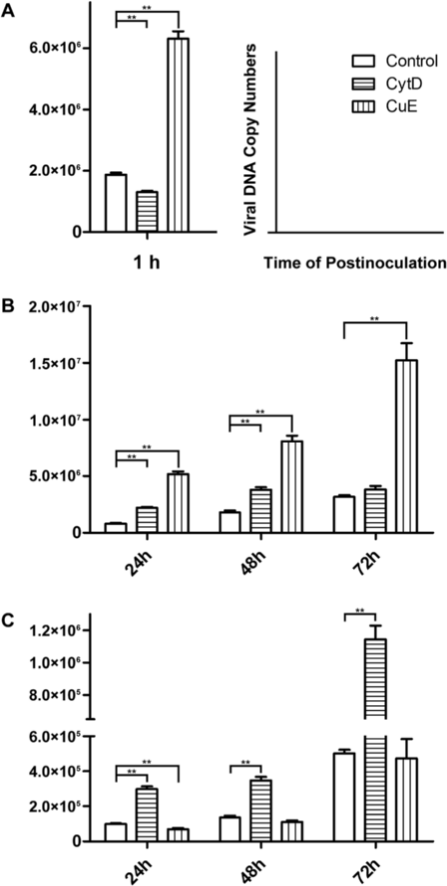
**Viral lifecycle changes after CytD/CuE treatment. (A)** Effect of microfilament depolymerisation and polymerization on viral invasion. To modify microfilaments, IPEC-J2 cells were treated with CytD or CuE for 2 h. Cells were then infected with PCV2 at 3 × 10^2.5^ TCID_50_/ml for 1 h and harvested. Viral DNAs were quantitated by qRT-PCR. **(B)** Effect of microfilament depolymerisation and polymerization on viral replication. IPEC-J2 cells were infected with PCV2 at 3 × 10^2.5^ TCID_50_/ml. To modify microfilaments, CytD or CuE was added at 1 h p.i., and supernatants and cells were harvested at 24, 48 and 72 h p.i. respectively. Viral DNAs in supernatants and cells were quantitated by qRT-PCR assay. **(C)** Effect of microfilament depolymerisation and polymerization on viral release. IPEC-J2 cells were infected with PCV2 at 3 × 10^2.5^ TCID_50_/ml. To modify microfilaments, CytD or CuE was added at 1 h p.i., and supernatants were harvested at 24, 48 and 72 h p.i. respectively. Viral DNAs in supernatants were quantitated by qRT-PCR. Asterisks above columns indicate highly significant differences between groups (*P* <0.01) (n = 3).

To investigate the relationship between microfilament structure and the PCV2 life cycle, IPEC-J2 cells were treated with one of two chemical inhibitors of F-actin dynamics, CytD or CuE. In the first experiment, cells were pretreated with inhibitor for two hours and then infected with PCV2 for a short period to detect changes affecting viral invasion. At 1 h p.i. the viral copy number in CytD-treated cells decreased (*P* <0.01) relative to untreated controls, while copy number in CuE-treated cells increased (*P* <0.01) (Figure 4A). In a second experiment, cells were infected with PCV2 and then treated with inhibitor 1 h p.i. to examine impacts on viral replication. Viral copy number in CytD-treated cells increased significantly (*P* <0.01) at 24 and 48 h p.i. (Figure 4B). CuE treatment resulted in copy number increases as well (*P* <0.01) at 24, 48 and 72 h p.i. (Figure 4B). In the final experiment, cells were treated as described in experiment 2, but viral copy number was assayed in the culture medium after cells had been removed. Viral copy number increased (*P* <0.01) at 24, 48 and 72 h p.i. after CytD treatment (Figure 4C). In contrast, viral copy number decreased (*P* <0.01) at 24 h p.i. after CuE treatment (Figure 4C).

## Discussion

Enteritis is one manifestation of PCV2 infection. The clinical signs are diarrhoea, which is initially yellowish but progresses to black, accompanied by retardation of growth [8]. The most consistent and predominant histopathological features of PCV2-associated enteritis are granulomatous inflammation and lymphoid depletion in the Peyer’s patches in the small and large intestines, and the presence of intra-cytoplasmic inclusion bodies [12]. Investigations of enteritis in swine have been performed in animal infection models, but thus far none have been conducted *in vitro* using porcine intestinal cell lines. We therefore selected the IPEC-J2 cell line, a permissive host for enteric pathogens with typical epithelial cell characteristics [27] for this study.

PCV2 infection causes ultra-structural changes in the mitochondria, affecting both overall shape and internal features, including matrix and cristae morphology. The diameter of porcine circovirus particles is about 17 nm, and virus particles with these dimensions were observed by TEM in infected IPEC-J2 cells. Immunofluorescence confirmed the presence of PCV2 inclusions as well.

We demonstrated conclusively that PCV2 can infect IPEC-J2 cells. PCV2 DNAs in infected IPEC-J2 cells increase steadily from 6 to 96 h p.i. but decrease at 120 h p.i., presumably due to the depletion of nutrients in the medium. Circoviruses depend on cellular polymerases for their replication [28], and PCV genomic DNA replication depends on cellular enzymes expressed during the S-phase of the host cell cycle [29].

**Figure 5:**
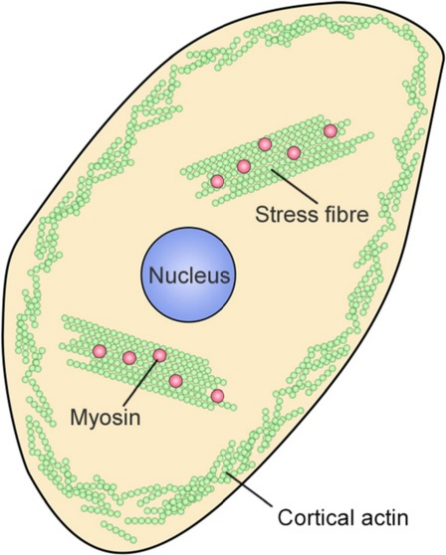
**Sketch map of microfilament.**

Within the cell, actin filaments can be arranged to form diverse structures. Stress fibres are large assemblies of actin filaments that can span the length of the cell (Figure 5). Cortical actin is a loosely organized network of actin filaments associated with the plasma membrane (Figure 5). Actin filaments also can be organized to produce a range of cellular extensions [30]. Viral infection is known to induce cytoskeletal reorganization. In this study, we found that PCV2 infection was accompanied by changes in microfilament organization at various time points post infection, possibly due to the production of new actin-based structures. Total F-actin in infected cells decreased at 24 and 48 h p.i. and then increased at 72 h p.i., compared to 1 h p.i. Cytoskeletal changes, monitored using microscopy, occurred in parallel, with a reduction in perinuclear microfilaments in PCV2-infected cells at 24 and 48 h p.i. At 72 h p.i., microfilaments were disorganized at the cell border but appeared to be reorganized in adjacent regions, suggesting that new actin-based structures had been produced. Since cells transition from normal to pathological states during viral infection, drastic changes in the regulation of cell signaling, cytoskeletal structure, and cell cycle are to be expected.

For viral invasion to occur, the physical barrier presented by cortical actin must be overcome. Virus binding initiates signaling events that change the cell surface and activate endocytosis [31], in which cytoskeletal actin plays an important role. Existing actin filaments undergo severing and depolymerization, while new actin filaments are polymerized from monomeric actin subunits and by branching from existing filaments [32]. CytD, which inhibits actin polymerization, strongly reduced PCV2 incursion in IPEC-J2 cells. In contrast, CuE treatment, which inhibits actin depolymerization, increased PCV2 invasion. These results indicate that actin polymerization may be required during PCV2 infection in IPEC-J2 cells. A similar requirement has been shown during PCV2 infection of monocytes/macrophages [33] and internalization into DCs [34].

Viral replication increased in both CytD and CuE-treated cells. Interestingly, CytD treatment enhanced viral replication while decreasing viral entry. This may be due to the fact that CytD not only disrupts actin microfilaments but also activates p53-dependent pathways, causing arrest of the cell cycle at the G1-S transition [35]. During the G1-S transition, chromosomes are duplicated by the cell, and PCV genomic DNA replication depends on cellular enzymes that are expressed during this period [29].

After CuE treatment, PCV2 replication increased significantly. Although a corresponding increase in viral release might be expected, our data showed no significant change in release compared with the control group. Currently, little is known about the egress of non-enveloped viruses from infected cells, commonly thought to occur as a virus burst after cell disintegration. *Vaccinia virus* (VV)-infected cells exhibit a unique phenotype involving changes in the actin cytoskeleton that are required for the spread of the infection [36]. In this system, CytD-mediated dissolution of cortical actin restores viral movement to the cell periphery in the absence of functional F11 (coded by the gene *F11L*), supporting the conclusion that VV reorganizes the cortical actin network to allow virus access to the plasma membrane. Our study found that PCV2 infection resulted in a loss of actin stress fibres and production of new actin-based structures at later stages of infection. We surmise that CytD treatment facilitates this process, causing CytD-treated cells to release more virus than untreated cells. In contrast, cells treated with CuE exhibit the opposite effect. Therefore, the dissolution of cortical actin may be required during PCV2 egress from IPEC-J2 cells.

## Conclusions

PCV2 not only infects IPEC-J2 cells but also proliferates in them, demonstrating that IPEC-J2 cells can serve as an intestinal cell infection model for studying PCV2 pathogenesis. When PCV2 invades IPEC-J2 cells, it causes actin polymerization, which may be conducive to viral invasion. At the replication and release phase, PCV2 appears to induce a reduction in stress fibres and production of new actin structures, leading us to conclude that the remodeling of cortical actin may facilitate viral release.
